# Enhancement of Transcription by a Splicing-Competent Intron Is Dependent on Promoter Directionality

**DOI:** 10.1371/journal.pgen.1006047

**Published:** 2016-05-06

**Authors:** Neha Agarwal, Athar Ansari

**Affiliations:** Department of Biological Sciences, Wayne State University, Detroit, Michigan, United States of America; The University of North Carolina at Chapel Hill, UNITED STATES

## Abstract

Enhancement of transcription by a splicing-competent intron is an evolutionarily conserved feature among eukaryotes. The molecular mechanism underlying the phenomenon, however, is not entirely clear. Here we show that the intron is an important regulator of promoter directionality. Employing strand-specific transcription run-on (TRO) analysis, we show that the transcription of mRNA is favored over the upstream anti-sense transcripts (uaRNA) initiating from the promoter in the presence of an intron. Mutation of either the 5′ or 3′ splice site resulted in the reversal of promoter directionality, thereby suggesting that it is not merely the 5′ splice site but the entire splicing-competent intron that regulates transcription directionality. ChIP analysis revealed the recruitment of termination factors near the promoter region in the presence of an intron. Removal of intron or the mutation of splice sites adversely affected the promoter localization of termination factors. We have earlier demonstrated that the intron-mediated enhancement of transcription is dependent on gene looping. Here we show that gene looping is crucial for the recruitment of termination factors in the promoter-proximal region of an intron-containing gene. In a looping-defective mutant, despite normal splicing, the promoter occupancy of factors required for poly(A)-dependent termination of transcription was compromised. This was accompanied by a concomitant loss of transcription directionality. On the basis of these results, we propose that the intron-dependent gene looping places the terminator-bound factors in the vicinity of the promoter region for termination of the promoter-initiated upstream antisense transcription, thereby conferring promoter directionality.

## Author Summary

Eukaryotic genes differ from their prokaryotic counterparts in having intervening non-coding sequences called introns. The precise biological role of introns in eukaryotic systems remains unclear even more than forty years after their initial discovery. One function of intron that has been remarkably conserved during evolution is their ability to enhance the transcription of genes that harbor them. How does the intron regulate transcription, however, is not known. Here we show that the intron enhances gene expression by affecting direction of the promoter-initiated transcription. In the presence of an intron, polymerase tends to transcribe the downstream coding region producing mRNA, while in the absence of a splicing-competent intron polymerase starts transcribing promoter upstream region producing upstream antisense RNA (uaRNA). Intron-mediated promoter directionality was dependent on gene looping, which is the interaction off the promoter and terminator region of a gene in a transcription-dependent manner. We show that the intron-dependent gene looping facilitates the recruitment of termination factors in the promoter-proximal region. The recruited termination factors stop uaRNA synthesis thereby conferring directionality to the promoter-bound polymerase.

## Introduction

Although introns were discovered more than four decades ago, their precise physiological role in biological systems still remains an enigma [1, 2]. One of the evolutionarily conserved functions of introns in eukaryotes is in regulation of the mRNA output of a gene [2–6]. The promoter-proximal introns often stimulate transcription of genes that harbor them [4, 7–13]. This phenomenon of enhancement of transcription by a splicing-competent intron is called ‘intron-mediated enhancement of transcription’ (IME) [1, 2, 4, 6, 14].The discovery of IME coincided with the development of cDNA technology. It was observed that the expression of the cDNA version of a gene is much less efficient than its native intron-containing counterpart in transfected mammalian cell lines [12, 15]. It was soon realized that the effect of an intron on transcription is a general feature of all eukaryotic organisms, including yeast, flies, worms, plants and humans [4]. Despite the ubiquity, the molecular mechanism underlying the phenomenon remains elusive even more than 25 years after its initial discovery.

Although less than 5% of genes in budding yeast contain introns, the intron-containing genes contribute nearly 28% of mRNA produced in yeast cells [3, 16]. We previously demonstrated that the intron-mediated enhancement of transcription in yeast involves gene looping, which is the physical interaction of the promoter and terminator regions of a gene in a transcription-dependent manner [10]. How the intron-facilitated looped gene architecture brings about enhancement of transcription, however, was not clear. A clue came when our laboratory and others demonstrated that gene looping confers directionality to the promoter-initiated transcription [17, 18]. The eukaryotic promoters and terminators are generally located in nucleosome free regions. Genomewide analysis has revealed that the promoters of most RNAPII-transcribed genes are bidirectional [19–27]. The transcription initiates in both the sense and upstream antisense directions from these promoters. Transcription in sense direction produces mRNA, while upstream antisense transcription generates non-coding transcripts called uaRNA (upstream antisense RNA) or PROMPT (Promoter upstream transcript) [19, 21, 26]. Transcription of the mRNA continues till the polymerase reaches the 3′ end of the gene, whereas synthesis of uaRNA is terminated when the transcript is just a few hundred to a thousand kilobase long [20]. This phenomenon is referred to as ‘promoter directionality’ [28]. It is generally believed that the uaRNA synthesis in yeast is terminated in a poly(A)-independent manner by the Nrd1-Nab3-Sen1 complex [29, 30]. In mammalian systems, however, uaRNA transcription is terminated by the same cleavage and polyadenylation machinery that stops mRNA synthesis at the 3′ end of a gene in a poly(A)-dependent manner [19, 21]. A number of reports suggest that the components of cleavage and polyadenylation machinery are involved in the termination of yeast uaRNA transcription as well [17, 18, 29–32]. In both yeast and higher eukaryotes, uaRNA is immediately degraded by the RNA surveillance machinery. Because of their short half-life, yeast uaRNA species are often referred to as cryptic unstable transcripts (CUTs) [23, 24]. In mammalian cells, the asymmetric distribution of poly(A) site and U1 snRNA-binding sites in the promoter-proximal region is believed to contribute to transcription directionality [19, 21]. In budding yeast, however, gene looping has been shown to confer promoter directionality [17, 18]. How gene looping enhances transcription directionality, however, is not clear.

Here we show that the transcription directionality of a subset of genes in yeast is dependent on the presence of a splicing-competent intron. The intron facilitates the recruitment of CF1, CPF and Rat1 termination complexes in the vicinity of the promoter region. We provide evidence that the intron-dependent gene looping facilitates the recruitment of termination factors near the promoter region. The recruited termination factors selectively terminate uaRNA synthesis, thereby conferring directionality to the promoter-initiated transcription.

## Results

### Introns confer promoter directionality

Research conducted during last eight years has confirmed that the nucleosome free region located at the 5′ end of most RNAPII-transcribed genes contains two unidirectional promoters [25, 26]. Each of these promoters assembles its own preinitiation complex (PIC) and is competent to initiate transcription [25, 27]. Mechanisms are in place in the cell to limit upstream antisense transcription and promote transcription in the sense direction. In mammalian systems, asymmetric distribution of 5′ splice sites and poly(A) sites in the promoter-proximal region has been shown to play a crucial role in conferring promoter directionality [19, 21]. The presence of 5′ splice sites in the promoter downstream region inhibits poly(A)-dependent termination of transcription, while the absence of 5′ splice sites in the promoter upstream region allows poly(A)-dependent termination of transcription in that region. The net result of this arrangement is that the synthesis of mRNA is favored over uaRNA. There is no such asymmetric distribution of U1 binding sites near yeast promoters [30, 33], but a subset of yeast genes contain introns. Since a promoter-proximal intron enhances transcription of mRNA, we hypothesized that the intron-mediated enhancement of mRNA synthesis could be, at least in part, due to the effect of the intron on promoter directionality.

To test this hypothesis, we examined transcription of three intron-containing genes, *IMD4*, *ASC1* and *APE2*, in the promoter-proximal upstream antisense and downstream sense direction in the presence and absence of an intron. We constructed strains with the intron-less version of these three genes following the strategy described in Moabbi et al., (2012) [10]. The mRNA and uaRNA levels were then compared by reverse transcription-polymerase chain reaction (RT-PCR) in the presence and absence of the intron. RT-PCR analysis revealed that the mRNA level of *IMD4*, *ASC1* and *APE2* deceased by 2.5 to 10 fold upon deletion of the intron (S1B Fig). In contrast, the uaRNA content of all three genes registered an increase in the absence of intron. The uaRNA level of *APE2* increased by about 15 fold, while that of *ASC1* and *IMD4* by about 1.6 fold upon deletion of intron (S1B Fig). These results suggested that the intron could be playing a role in regulating the direction of promoter-initiated transcription. The presence of an intron favored synthesis of mRNA over uaRNA, while the absence of intron switched direction of transcription so as to favor the uaRNA synthesis.

**Fig 1:**
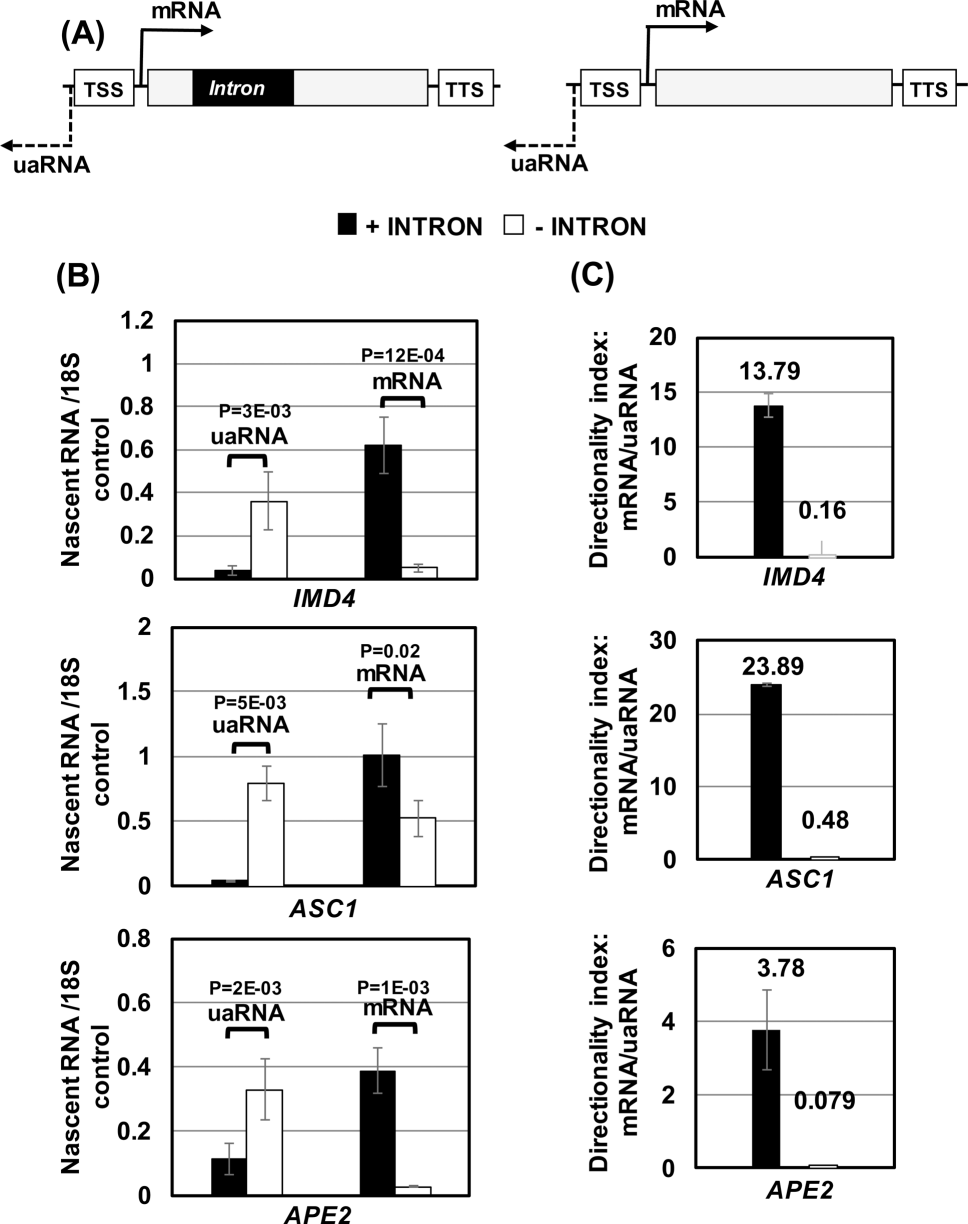
Intron regulates promoter directionality. (A) Schematic depiction of a gene with and without intron. TSS represents transcription start site, and TTS represents transcription termination site. (B) Quantification of TRO analysis of *IMD4*, *ASC1* and *APE2* in the presence (black bars) and absence of intron (white bars) to detect the expression of mRNA or uaRNA. The transcript level of 18S was used as a control for normalization. P values were calculated by two-tailed student t-test. (C) Directionality indices of *IMD4*, *ASC1* and *APE2* in the presence (black bars) and absence of intron (white bars).

There was, however, a possibility that the observed alteration in the steady state level of mRNA and uaRNA was not due to the effect of intron on transcription, but on the stability of transcripts. The presence of intron could somehow stabilize mRNA, but facilitate the degradation of uaRNA by exosomes. To rule out this possibility, we performed strand-specific transcription run-on (TRO) analysis as described in Medler and Ansari (2015) [34]. Briefly, the technique involved labeling the nascent transcripts with Br-UTP, purifying Br-UTP labeled RNA using anti-Br-UTP affinity beads, and then subjecting affinity purified nascent RNA to RT-PCR analysis as described above. Strand-specific TRO analysis revealed about a 2 to10 fold decrease in nascent transcription of *IMD4*, *ASC1* and *APE2* in the sense direction (mRNA synthesis) upon deletion of intron (Fig 1B). Simultaneously, there was about a 3–10 fold increase in upstream antisense transcription (uaRNA synthesis) in the absence of the intron (Fig 1B). These results confirmed the findings observed above in (S1 Fig), and corroborated the role of the intron in promoter directionality for the three genes examined here.

To gain an insight into the role of the intron in promoter directionality, we calculated the directionality index by dividing nascent mRNA level with nascent uaRNA level for each tested gene in the presence and absence of an intron. The directionality indices in the presence of intron for these three genes ranged from 4 to 25 (Fig 1C). Upon deletion of the intron, the directionality index registered a decline by about 50–250 fold (Fig 1C).

### Intron-dependent transcription directionality requires a splicing-competent intron

**Fig 2:**
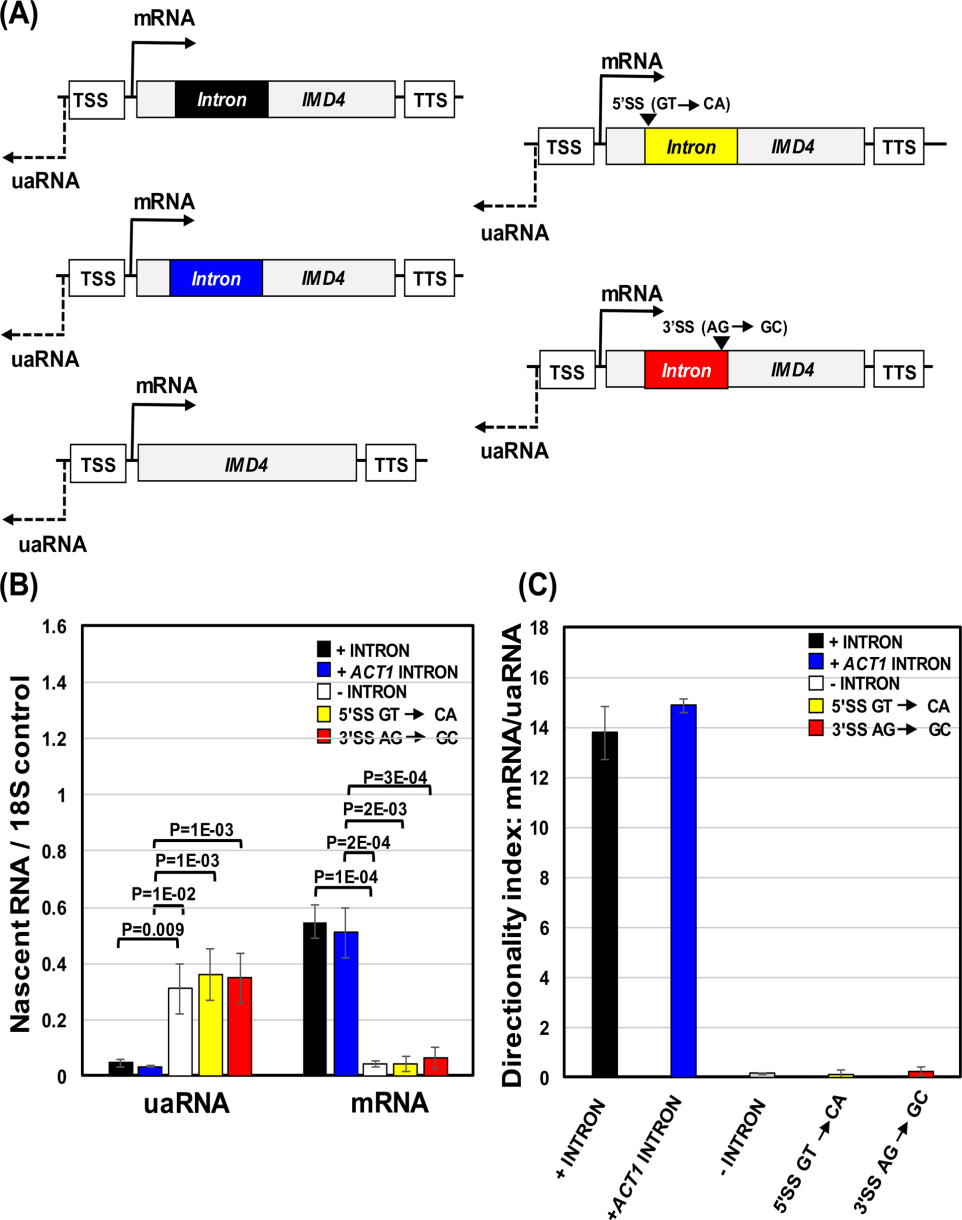
Intron-dependent transcription directionality requires a splicing-competent intron. (A) Schematic depiction of *IMD4* with a native wild type intron, with a wild type *ACT1* intron, without intron, a 5′ splice-site (5′SS) mutated *ACT1* intron and a 3′ splice-site (3′SS) mutated *ACT1* intron indicating the direction of transcription of mRNA and promoter-initiated uaRNA. TSS represents transcription start site, and TTS represents transcription termination site. (B) Strand specific TRO analysis of *IMD4* with a native wild type intron (black bars), with a wild type *ACT1* intron (blue bars), without intron (white bars), a 5′ splice-site (5′SS) mutated *ACT1* intron (yellow bars) and a 3′ splice-site (3′SS) mutated *ACT1* intron (red bars) to detect the expression of mRNA and uaRNA. The transcript level of 18S was used as a normalization control. P values were calculated by two-tailed student t-test. (C) Directionality indices of *IMD4* with native wild type intron (black bar), with wild type *ACT1* intron (blue bar), without intron (white bar), a 5ꞌ splice-site (5ꞌSS) mutated intron (yellow bar), and a 3ꞌ splice-site (3ꞌSS) mutated intron (red bar).

Having demonstrated the role of intron in promoter directionality in budding yeast, we next asked if it is just the 5′ splice site as has been shown in mammalian systems or the whole splicing-competent intron that confers directionality to the promoter-initiated transcription in yeast cells. We therefore inserted a wild type, a 5′ splice site mutated, and a 3′ splice site mutated *ACT1* intron into an intron-less *IMD4* gene as previously described (Moabbi et al., 2012) [10]. The 5′ splice site was mutated from GT to CA, while 3′ splice site region was mutated from AG to GC (Moabbi et al., 2012) [10]. Both mutations abolished splicing as a longer mRNA was produced (S2B Fig). The strand-specific TRO analysis was then carried out to detect transcription of mRNA and uaRNA of *IMD4* in the presence of a wild type native intron, in the presence of a wild type *ACT1* intron, in the absence of an intron, in the presence of 5′ splice site mutated and a 3′ splice site mutated *ACT1* intron. The insertion of a wild type *ACT1* intron brought about a 10-fold increase in transcription of mRNA (Fig 2B). The enhancement of *IMD4* transcription by the *ACT1* intron was almost to the extent conferred by its native intron. As expected, the mutation of either the 5′ or 3′ splice site failed to enhance transcription of *IMD4* (Fig 2B). Simultaneously, we compared transcription of uaRNA. There was little detectable uaRNA signal in the presence of the native or wild type *ACT1* intron (Fig 2B). A 6–8 fold increase in nascent uaRNA signal was observed in the presence of a 5′ splice site mutated intron (Fig 2B). The mutation of the 3′ splice site gave similar results (Fig 2B). The drop in directionality index upon mutation of the 5′ or 3′ splice site was almost to the same extent (70–100 fold) as in the absence of an intron (Fig 2C). A logical conclusion of these results is that it is not the 5′ splice site alone, but the whole splicing-competent intron that confers transcription directionality to a subset of yeast genes.

### Intron facilitates the recruitment of termination factors to the promoter-proximal region

In mammalian cells, uaRNA synthesis is terminated in a poly(A)-dependent manner by the cleavage and polyadenylation machinery [19, 21]. In contrast, promoter-initiated upstream antisense transcription in yeast is believed to be terminated by the Nrd1-Nab3-Sen1 complex in a poly(A)-independent manner [29, 30]. We hypothesized that the presence of a splicing-competent intron facilitates the recruitment of termination factors in the vicinity of the promoter region. The recruited termination factors stop upstream antisense transcription, thereby providing directionality to the promoter-initiated transcription. To test this hypothesis, we examined the recruitment of termination factors in the promoter-proximal region of a gene in the presence and absence of an intron. Although uaRNA in yeast belongs to the category of CUTs, which are predominantly terminated by the Nrd1-Nab3-Sen1 complex, the recent studies have also implicated CPF subunit Ssu72 and CF1 subunit Pcf11 in the termination of uaRNA transcription [17, 29, 31, 32]. We therefore checked for the presence of all four termination complexes; CF1, CPF, Rat1 and Nrd1 complexes, in the promoter-proximal region of *IMD4* and *ASC1* genes by ChIP as described in Al Husini et al., (2013) [18]. The termination factor ChIP was performed in strains with intron-containing or intron-less versions of the gene under investigation. The promoter occupancy of CPF complex was monitored in terms of recruitment of its Pta1 subunit, while CF1 complex recruitment was detected using its Rna15 subunit. Similarly, Rat1 complex recruitment was monitored using its Rat1 subunit, and Nrd1 complex was tracked using its Nab3 subunit. The strains carrying epitope-tagged version of these termination factors were generated to facilitate ChIP.

**Fig 3:**
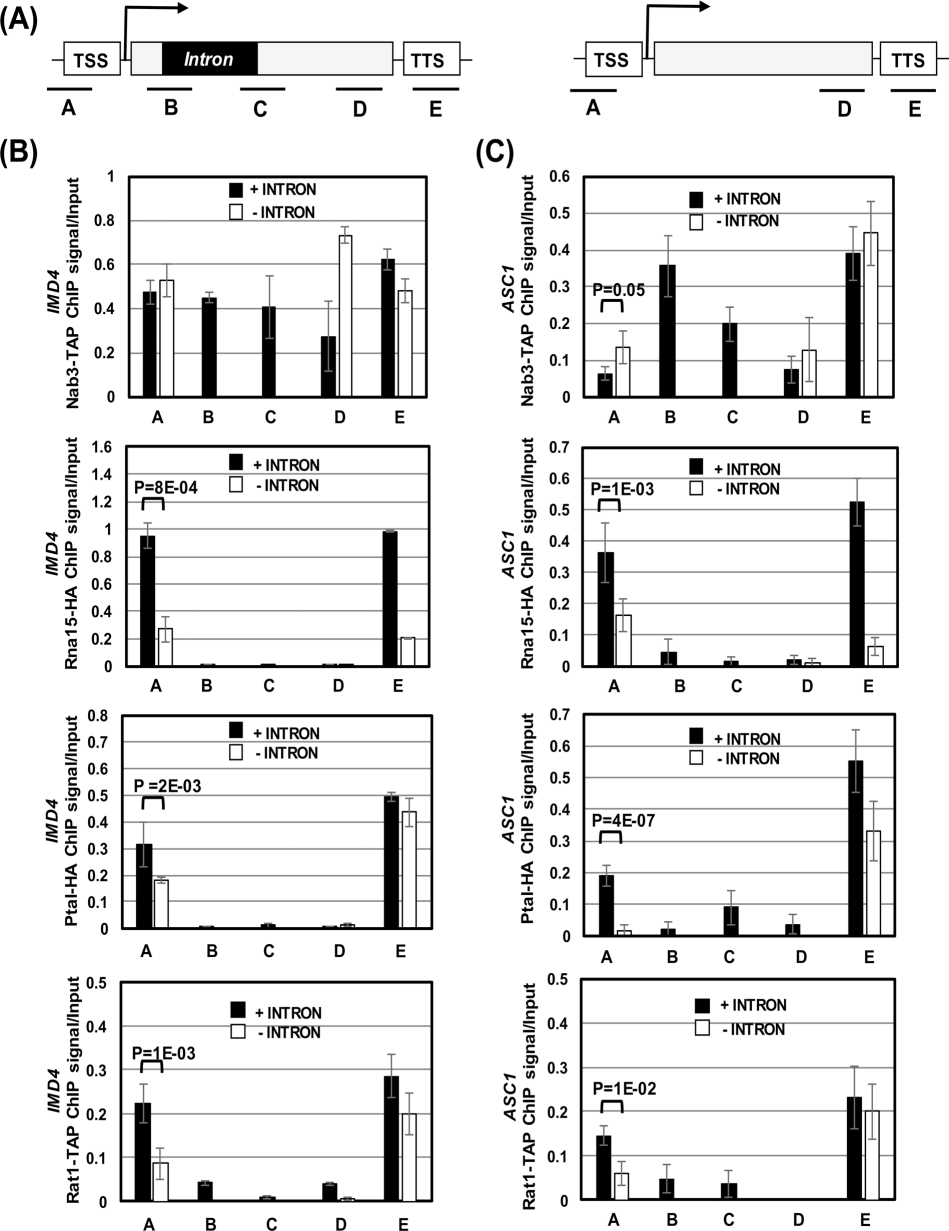
Intron facilitates the recruitment of termination factors near the promoter-proximal region. (A) Schematic depiction of a gene with and without intron indicating the position of ChIP primer pairs A, B, C, D, and E. TSS represents transcription start site, and TTS represents transcription termination site. (B, C) Quantification of ChIP results showing crosslinking of termination factors Nab3, Rna15, Pta1 and, Rat1 to different region of *IMD4* and *ASC1* in the presence (black bars) and absence (white bars) of intron. Input represents DNA prior to immunoprecipitations. P value was calculated as described previously.

We first examined the recruitment of Nrd1 complex subunit Nab3 at *IMD4* and *ASC1* in the presence and absence of an intron. Nab3 was recruited at both the 5′ and 3′ ends of *IMD4* with almost equal intensity in the presence of an intron (Fig 3B). Upon deletion of intron, there was no appreciable change in the Nab3 occupancy of either the 5′ or the 3′ end of *IMD4* (Fig 3B). Although Nab3 crosslinking to the 5′ end of *ASC1* was about 60% less than that at the 3′ end of the gene, still no significant change in Nab3 crosslinking was observed at the 5′ end of gene in the absence of the intron (Fig 3C). These results suggest that the recruitment of the Nrd1 complex at the 5′ end of *IMD4* and *ASC1* genes is not dependent on the presence of an intron. We then checked for the recruitment of CF1, CPF and Rat1 complexes in the vicinity of the promoter of *IMD4* and *ASC1* by ChIP. The CF1 subunit Rna15 was found crosslinked to both the ends of *IMD4* and *ASC1* in the presence of an intron (Fig 3B and 3C). These results are in agreement with our published results that the CF1 complex occupies distal ends of a number of yeast genes in a transcription-dependent manner [18, 34, 35]. In the absence of an intron, however, both the 5′ and 3′ occupancy of Rna15 decreased. The Rna15 signal at the promoter of *IMD4* and *ASC1* decreased by about 3.5 fold and 2.2 fold respectively upon deletion of the intron (Fig 3B and 3C). A similar reduction in the promoter occupancy of Pta1, which is a subunit of CPF complex, and Rat1 was observed for both *IMD4* and *ASC1* in the absence of an intron (Fig 3B and 3C). The promoter crosslinking of Pta1 decreased by about 2–12 fold, and that of Rat1 by about 2–4 fold in the intron-less versions of these two genes (Fig 3B and 3C). The overall conclusion of these results is that the recruitment of CF1, CPF and Rat1 cleavage-polyadenylation/termination complexes at the 5′ end of *IMD4* and *ASC1* occurs in an intron-dependent manner. Furthermore, the promoter occupancy of these termination complexes coincides with the enhanced directionality of promoter-initiated transcription. A corollary of these observations is that the intron-dependent recruitment of termination factors near the 5′ end of genes could be playing a critical role in transcription directionality.

### Recruitment of termination factors at the promoter confers directionality

**Fig 4:**
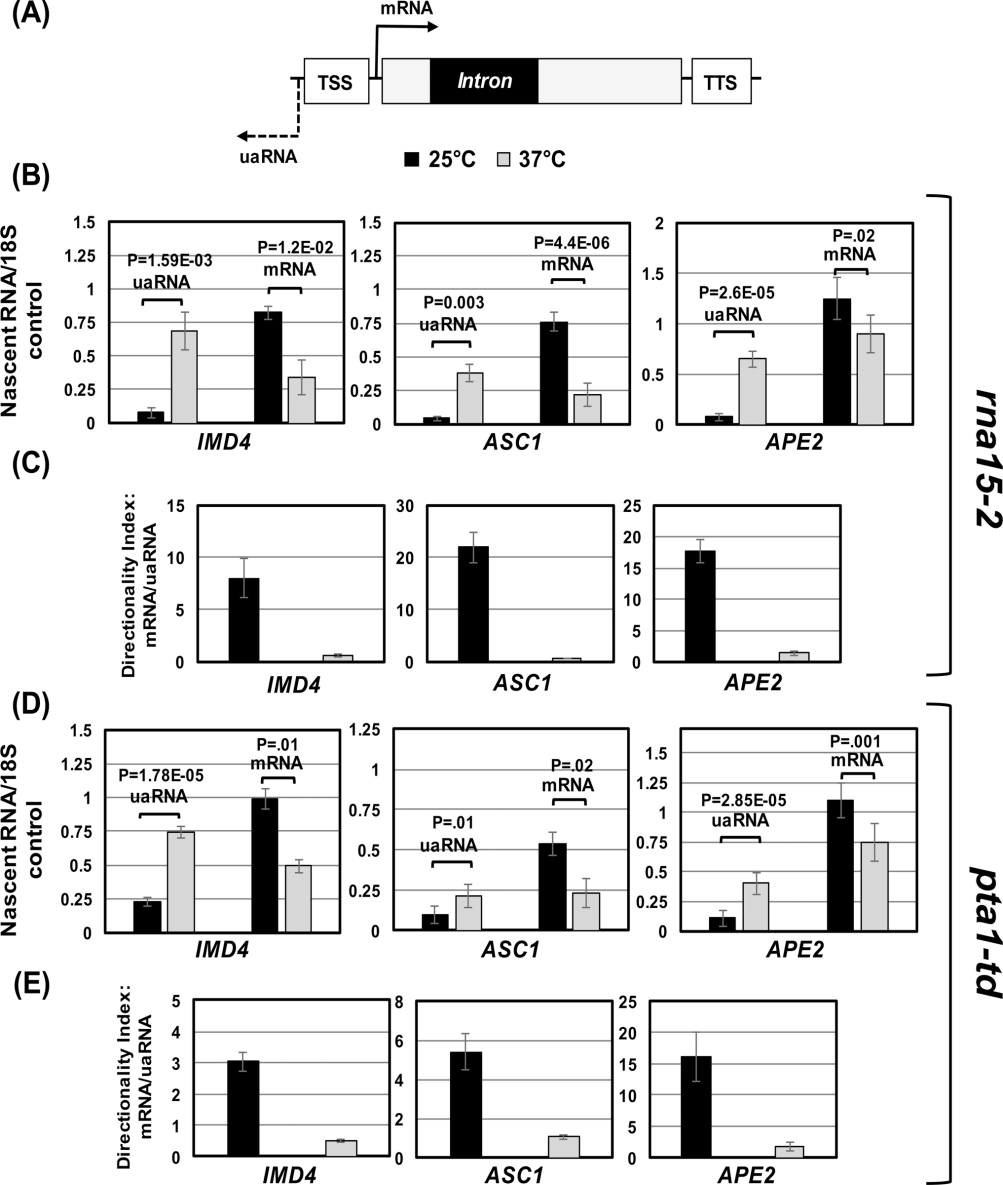
Inactivation of termination factors affects uaRNA and mRNA transcription as well as transcription directionality. (A) Schematic depiction of a gene with intron. TSS represents transcription start site, and TTS represents transcription termination site. (B) Quantification of TRO analysis of *IMD4*, *ASC1* and *APE2* in *rna15-2* mutant at 25°C (black bars) and 37°C (grey bars) to detect the expression of mRNA or uaRNA. The transcript level of 18S was used as a control for normalization. P values were calculated by two-tailed student t-test. (C) Directionality indices of *IMD4*, *ASC1* and *APE2* in *rna15-2* mutant at 25°C (black bars) and 37°C (grey bars). (D) Quantification of TRO analysis of *IMD4*, *ASC1* and *APE2* in *pta1-td* mutant at 25°C (black bars) and 37°C (grey bars) to detect the expression of mRNA or uaRNA. The transcript level of 18S was used as a control for normalization. P values were calculated by two-tailed student t-test. (E) Directionality indices of *IMD4*, *ASC1* and *APE2* in *pta1-td* mutant at 25°C (black bars) and 37°C (grey bars).

The experiments described above clearly demonstrated an increase in the promoter recruitment of factors required for poly(A)-dependent termination of transcription in the presence of an intron. It was, however, not clear if the recruited termination factors were enhancing transcription directionality by affecting uaRNA transcription. We therefore examined nascent uaRNA and mRNA levels in the promoter-proximal region of *IMD4*, *ASC1* and *APE2* in temperature-sensitive mutants of *RNA15* (*rna15-2*) and *PTA1* (*pta1-td*) by strand-specific TRO approach. The results show that uaRNA transcription increased by about 5-fold upon shifting of *rna15-2* cells to elevated temperature (Fig 4B). A similar increase in nascent uaRNA level was observed when *pta1-td* cells were shifted to non-permissive temperature (Fig 4D). The increase in uaRNA level in *pta1-td* mutant, however, was to a lesser extent (about 3-fold). No such increase in uaRNA transcription was observed in the isogenic wild type cells at elevated temperature (S3B Fig). In contrast, mRNA transcription registered a decline upon shifting of mutants to 37°C for all three genes (Fig 4B and 4D). The enhanced uaRNA synthesis in *rna15-2* and *pta1-td* mutants at elevated temperature was accompanied by a concomitant decrease in directionality indices (5–10 fold) (Fig 4C and 4E). These experiments strongly suggest that the termination factors at the promoter are enhancing transcription directionality by preventing uaRNA transcription.

### Gene looping facilitates the recruitment of termination factors near the 5′ end of genes

**Fig 5:**
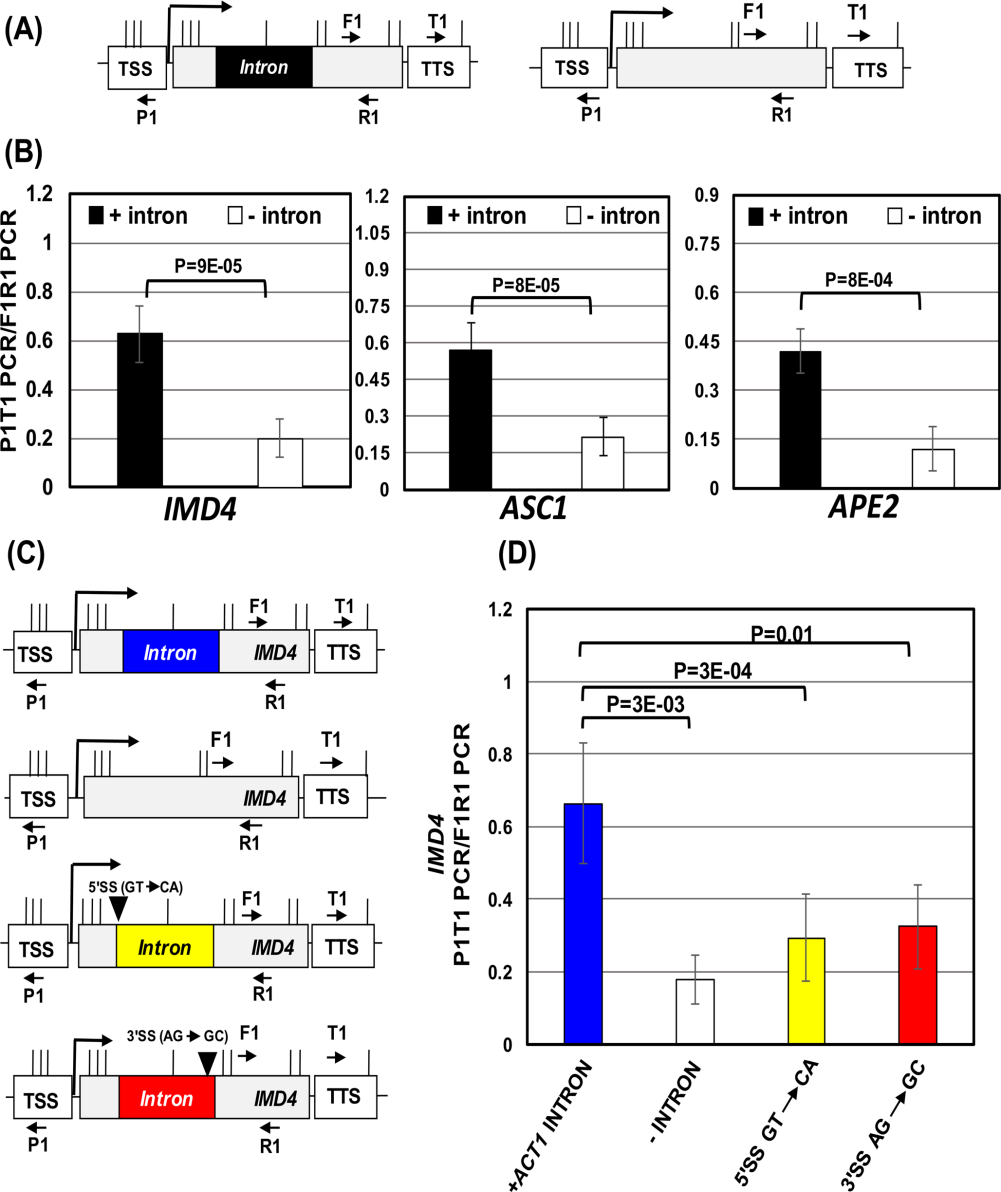
Gene looping is reduced upon deletion or mutation of the 5′ or 3′ splice site of an intron. (A) Schematic depiction of a gene with a native wild type intron and without intron indicating position of restriction sites (vertical lines) and primers P1-T1 and F1-R1 (arrows) used in CCC assay. TSS represents transcription start site, and TTS represents transcription termination site. (B) Quantification of CCC results of *IMD4*, *ASC1* and *APE2* genes in the presence of their native wild type intron (black bars) and in the absence of intron (white bars). P1-T1 PCR indicates gene looping, while F1-R1 PCR is the loading control indicating that an equal amount of template DNA was used in each CCC reaction. (C) Schematic depiction of *IMD4* gene with a wild type *ACT1* intron, without intron, a 5ꞌ splice-site (5ꞌSS) mutated *ACT1* intron and a 3ꞌ splice-site (3ꞌSS) mutated *ACT1* intron indicating position of restriction sites (vertical lines) and primers P1-T1 and F1-R1 (arrows) used in CCC assay. (D) Quantification of CCC results of *IMD4* in the presence of a wild type *ACT1* intron (blue bar), without intron (white bar), presence of a 5ꞌ splice-site (5ꞌSS) mutated *ACT1* intron (yellow bar) and a 3ꞌ splice-site (3ꞌSS) mutated *ACT1* intron (red bar). P1-T1 PCR indicate gene looping, while F1-R1 PCR is the loading control indicating that an equal amount of template DNA was used in each CCC reaction.

Next we asked how the presence of an intron facilitates the recruitment of termination factors in the promoter-proximal region. A clue came from our previous observation that a gene assumes a looped conformation in the presence of an intron (Moabbi et al., 2012) [10]. A gene loop is formed due to the physical interaction of the terminator region of a gene with its cognate promoter in a transcription-dependent manner [36]. Gene looping has been shown to affect promoter directionality in budding yeast [17, 18]. We hypothesized that it is the looped gene structure formed in the presence of a splicing-competent intron that facilitates the recruitment of terminator-bound factors to the promoter end of a gene owing to the close physical proximity of the promoter and terminator regions. We have already demonstrated the intron-dependent gene looping of *INO1* and *ASC1* [10], but it was not clear if *IMD4* and *APE2* also exhibit a similar intron-dependent change in gene conformation. We therefore performed ‘Chromosome Conformation Capture’ (CCC) analysis of *IMD4*, *ASC1* and *APE2* in the presence and absence of their native wild type intron in the same batch of cells that were used for measuring transcription directionality in Fig 1 above, following the protocol described in El Kaderi et al., (2012) [37]. The CCC assay measures gene looping in terms of a PCR product obtained using P1T1 primer pair that flanks the promoter and terminator regions as shown in Fig 5A. A robust P1T1 looping signal was observed for *IMD4*, *ASC1* and *APE2* in the presence of the native wild type intron (Fig 5B). The looping signal decreased by about 3 fold in the absence of an intron (Fig 5B). Thus, the promoter-proximal recruitment of termination factors at all three genes used in this analysis was accompanied by the gene assuming a looped architecture.

**Fig 6:**
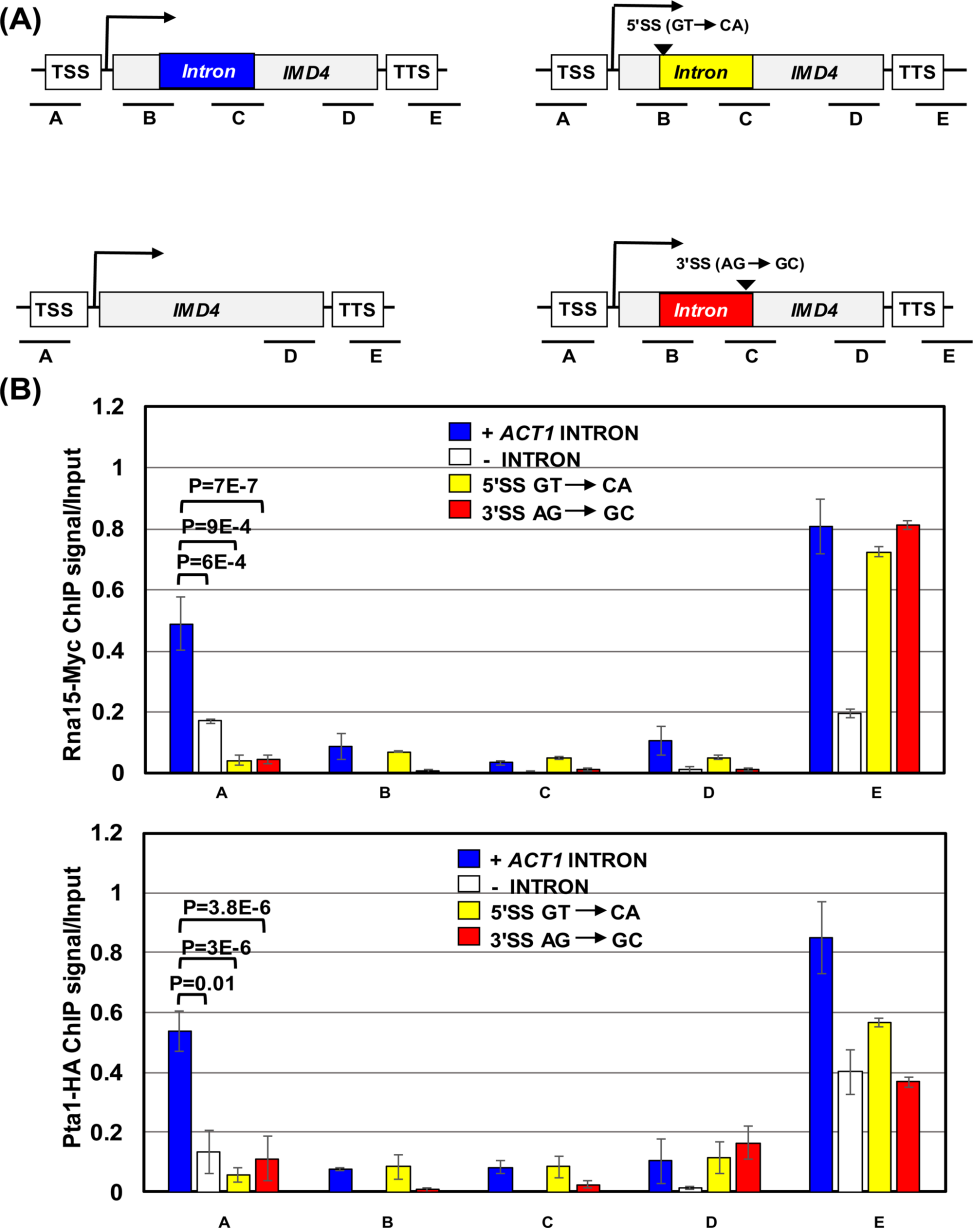
Splicing competent intron is required for the recruitment of termination factor near the promoter-proximal region. (A) Schematic depiction of *IMD4* genes with a wild type *ACT1* intron, without intron, a 5ꞌ splice-site (5ꞌSS) mutated *ACT1* intron and a 3ꞌ splice-site (3ꞌSS) mutated *ACT1* intron indicating the position of ChIP primer pairs A, B, C, D, and E. TSS represents transcription start site, and TTS represents transcription termination site. (B) Quantification of ChIP results showing crosslinking of termination factors Rna15 and Pta1 to different region of *IMD4* with a wild type *ACT1* intron (blue bars), without intron (white bars), a 5ꞌ splice-site (5ꞌSS) mutated intron (yellow bars) and a 3ꞌ splice-site (3ꞌSS) mutated intron (red bars). Input represents DNA prior to immunoprecipitation.

Intron-dependent gene looping, however, is different from transcription-dependent looping of non-intronic genes. It is characterized by additional interactions of the intron with gene ends and requires functional 5′ and 3′ splice sites [10]. To corroborate the role of intron-dependent gene looping in the promoter-recruitment of termination factors, we measured conformation of *IMD4* gene in the presence of a wild type, a 5′ splice site mutated as well as a 3′ splice site mutated *ACT1* intron. A robust P1T1 looping signal was observed for *IMD4* in the presence of the wild type *ACT1* intron, which was almost to the same extent as in the presence of the native intron (Fig 5D). Mutation of either the 5′ or 3′ splice sites resulted in a decrease in looping signal, almost to the same extent as in the absence of intron (Fig 5D). We reasoned that if gene looping was responsible for the recruitment of termination factors at the promoter region of intron-containing genes, then loss of looping upon mutation of either 5′ or 3′ splice site will adversely affect the promoter occupancy of termination factors. ChIP analysis revealed that the promoter occupancy of CF1 subunit Rna15 and CPF subunit Pta1 was indeed reduced in the splice site mutants of *IMD4*. The promoter Rna15 signal was reduced by about 9–12.5 fold, while that of Pta1 declined by 5–10 fold in the splice site mutants (Fig 6B). The correlative nature of the gene looping and the promoter occupancy of termination factors support the idea that the looped gene architecture could be playing a crucial role in the loading of termination factors to the 5′ end of yeast genes.

**Fig 7:**
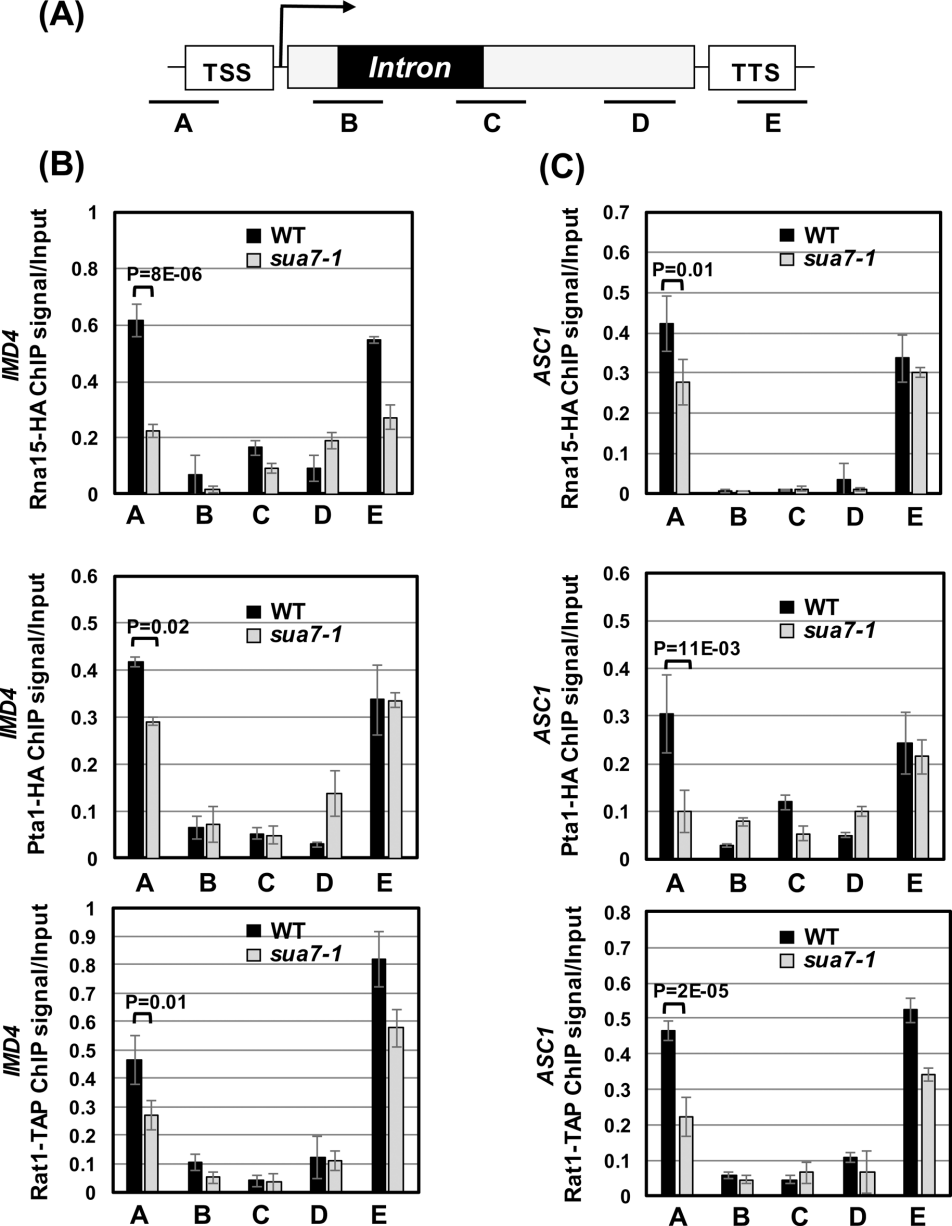
Gene looping is required for the recruitment of termination factors near the promoter-proximal region. (A) Schematic depiction of an intron-containing gene indicating the position of primer pairs A, B, C, D, and E used in ChIP assay. TSS represents transcription start site, and TTS represents transcription termination site. (B, C) Quantification of ChIP results showing crosslinking of termination factors Rna15, Pta1 and, Rat1 to different region of *IMD4* and *ASC1* in the wild type (black bars) and looping defective *sua7*-1 (grey bars)

To further explore the role of gene looping in the promoter recruitment of termination factors, we examined the promoter occupancy of termination factors in the looping-defective *sua7-1* strain. The *sua7-1* is an allele of the general transcription factor TFIIB with the glutamic acid at position 62 replaced with lysine (E62K) [38]. We have previously used this strain to show the role of gene looping in the intron-mediated enhancement of transcription [10]. This strain has also been used to demonstrate the role of gene looping in transcription memory and termination of transcription [34, 39–41]. We expected that if gene looping was responsible for the recruitment of termination factors at the 5′ end of genes, then the crosslinking of termination complexes in the promoter-proximal region will be compromised in the looping defective strain. We found that the promoter occupancy of all three termination complexes, CF1, CPF and Rat1, registered a decline in the looping defective mutant. The promoter recruitment of CF1 subunit Rna15, CPF subunit Pta1 and Rat1 complex subunit Rat1 decreased by about 1.5–3.5 fold in the looping defective *sua7-1* strain (Fig 7B and 7C). To rule out the possibility of loss of promoter recruitment of termination factors in the looping defective strain being an indirect effect of defective splicing in the *sua7-1* strain, we examined splicing of *IMD4* and *ASC1* pre-mRNA. The looping defect did not affect the splicing efficiency of either *IMD4* or *ASC1* transcripts (S4B Fig). If it was the looping-mediated recruitment of termination factors that conferred promoter directionality, then we expected that the transcription directionality will be adversely affected in the looping-defective *sua7-1* cells. Our results show that the transcription directionality is indeed compromised in the *sua7-1* strain (S5B Fig). These findings strongly support the idea that gene looping determines promoter directionality by facilitating the recruitment of termination factors to the 5′ end of genes.

## Discussion

Our published results suggest that it is either the transcription activator or the presence of an intron that facilitates transcription-dependent gene looping [10, 42]. The non-intronic genes are dependent on gene specific transcription activators for gene looping [42]. The intron-containing genes, however, require a splicing-competent intron to assume a looped gene conformation during transcription [10]. The promoter directionality of RNAPII-transcribed genes in budding yeast has been shown to be dependent on gene looping [17, 18]. Employing looping defective mutants, it was demonstrated that there is an increase in synthesis of uaRNA at the expense of mRNA in the absence of gene looping. These experiments, however, were performed with genes that exhibited activator-dependent gene looping. Here we show that the intron-dependent gene looping, which is characterized by additional interaction of the promoter and terminator regions with the intron, also confers directionality to the promoter-bound polymerase. How gene looping conferred transcription directionality was, however, not clear from any of the previous studies [17, 18]. On the basis of results presented here, we suggest a possible molecular mechanism underlying the enhancement of transcription directionality by the looped gene architecture. We propose that the proximity of the promoter and terminator regions in the gene loop allows the terminated polymerase along with the termination factors to be released from the 3′ end in the vicinity of the promoter of the gene. This leads to an increase in the local concentration of the termination factors near the 5′ end of a gene. These termination factors can now be recruited by the polymerase engaged in upstream anti-sense transcription leading to termination of uaRNA synthesis. Our hypothesis is supported by multiple experimental analyses. First, we observed enhanced crosslinking of the components of the CF1, CPF and Rat1 termination complexes near the 5′ end of several genes in the presence of an intron when the gene is in looped conformation (Fig 3B and 3C). Second, the recruitment of termination factors in the promoter-proximal region was compromised in the looping defective *sua7-1* strain (Fig 7B and 7C). This mutant effects gene looping and transcription directionality without any adverse effect on splicing (S4 and S5 Figs). Third, the promoter occupancy of the termination factors exhibited a declining trend in the absence of an intron and in the presence of a mutated introns (Fig 6B). The mutation of splice sites in the intron selectively abolishes looping of the gene under investigation without any adverse effect on global gene looping [10]. It corroborates the finding with the looping defective *sua7-1* mutant, which affects gene looping on a genomewide scale and therefore can potentially have an indirect effect on promoter directionality. The overall conclusion of these results is that it is looped gene architecture that facilitates the recruitment of termination factors near the 5′ end of a gene, and the termination factors then terminate the transcription of uaRNA thereby conferring promoter directionality.

In mammalian cells, asymmetric distribution of poly(A) sites and U1-binding sites has been shown to influence the recruitment of termination factors in the promoter-upstream region, which in turn terminates uaRNA synthesis [19, 21]. The transcription directionality of the mammalian β-globin gene, however, was found to be compromised in a looping defective mutant of the gene [17]. This invokes the possibility of gene looping playing a similar role in the recruitment of termination factors in the promoter-proximal region of at least a subset of mammalian genes during transcription.

If gene looping enhances transcription directionality by facilitating the recruitment of termination factors in the promoter-proximal region, the next logical question is why the promoter-recruited termination factors selectively terminate uaRNA synthesis, while mRNA transcription continues unabated. We have already shown that the activator-dependent gene looping facilitates recycling of polymerase from the terminator to the promoter for transcription in sense direction [18]. The intron-dependent gene looping may have a similar effect. A logical question is why the promoter recruited polymerase in a looped gene tends to preferentially transcribe in the sense direction, while it is terminated in the upstream anti-sense direction. We hypothesize that the differential effect of termination factors on the promoter-initiated divergent transcription could be due to differential chromatin structure in the vicinity of the promoter region. It has been shown that the histone modification pattern in the regions upstream and downstream of the bidirectional promoter is markedly different. In mammalian cells, the promoter downstream region is characterized by H3K79 dimethylation [22], which is the mark of elongating polymerase. In contrast, the promoter upstream region is deficient in H3K79 dimethylation [22]. Furthermore, H3K4 is trimethylated in the promoter downstream sense direction, while the upstream antisense region is marked by H3K4 monomethylation [27]. A similar differential modification of H3K27 was recently reported around the bidirectional promoter region in a murine cell line [27]. The H3K27 was found preferentially acetylated in the promoter upstream region near the antisense transcription start site in a subset of bidirectional promoters in murine macrophages. These differential chromatin marks around the bidirectional promoter region may inhibit elongation of uaRNA transcript and facilitate their termination by the termination factors.

The emerging view is that a vast majority of RNAPII-transcribed genes in yeast and mammalian systems have bidirectional promoters. The regulation of promoter directionality is critical for optimal transcription of these gene. We show a novel role of introns in yeast in regulating promoter directionality through looping-mediated recruitment of termination factors at the promoter. Less than 5% of yeast genes contain introns. In contrast, a vast majority of genes in higher eukaryotes contain introns. It is, therefore, tempting to speculate that introns might have a similar mechanistic impact on transcription directionality in higher eukaryotes as well.

## Materials and Methods

### Yeast strains

Yeast strains used in this study are listed in S1 Table.

### Cell cultures

Cultures were started by inoculating 5 ml of YP-dextrose medium with colonies from a freshly streaked plate and grown at 30°C with gentle shaking. Next morning, overnight grown cultures were diluted (1:100) to appropriate volume and grown to A_600_ ~0.6. Equal number of cells were used for strand-specific RT-PCR, CCC, ChIP or strand-specific TRO assays. The *rna15-2* and *pta1-td* mutants were grown at permissive temperature (25°C) till A_600_ reached 0.5. Cells were then shifted to non-permissive temperature (37°C) for 90 minutes and processed for strand-specific TRO analysis.

### Capture chromosome conformation assay (CCC)

CCC experiments were performed as described previously [37]. The primers used for 3C analysis are shown in S2 Table. The enzyme used for chromatin digestion of *IMD4* gene were Alu1 and Dra1, were obtained from New England Biolabs. Each experiment was performed with at least four independently grown cultures. The P1T1 PCR signals were normalized with respect to F1-R1 PCR signals.

### Chromatin immunoprecipitation (ChIP)

ChIP experiments (crosslinking, cell lysis and isolation of chromatin) were performed as described previously [42]. Different strains were constructed by tagging subunits of all four termination complexes (CPF, CF1, Rat1 and Nrd1) as mentioned in S1 Table. Anti-HA antibodies were used to pull down HA-tagged subunits, were obtained from Thermo-scientific. Anti-Myc antibodies were used to pull down Myc-tagged subunits were obtained from Upstate Biotechnology, and IgG-Sepharose beads purchased from GE Healthcare were used to pull down TAP-tagged subunits. For ChIP analysis, primers used for ChIP PCR are shown in S2 Table. Each experiment was repeated with at least four independently grown culture.

### Transcription Analysis

Transcription analysis was performed by RT-PCR approach as described previously [35]. The primers used for RT-PCR analysis are shown in S2 Table.

### Strand-specific ‘Transcription Run-On’ (TRO) assay

The strand-specific ‘Transcription Run-On’ (TRO) assay was performed as described previously in [34]. The primers used for making cDNA and PCR for all the genes are mentioned in S2 Table.

### Quantification

The data shown in Figures is the result of at least four biological replicates. The quantification and statistical analysis was performed as described in [37]. Error bars represent one unit of standard deviation. P-values were calculated by two-tailed student t-test.

## Supporting Information

S1 Fig Intron regulates promoter directionality. (A) Schematic depiction of a gene with and without intron indicating the sense (mRNA) or the promoter-initiated upstream anti-sense (uaRNA) transcripts. TSS is transcription start site, and TTS indicates transcription termination site. (B) RT-PCR analysis of *IMD4*, *ASC1* and *APE2* in the presence (black bars) and absence of intron (white bars) to detect the expression of mRNA or uaRNA. The transcript level of 18S was used as a control for normalization.
(PDF)

S2 Fig Splicing of pre-mRNA is affected in the splice site mutant strains. (A) Schematic depiction of *IMD4* gene containing wild type *ACT1* intron, 5ꞌ splice site mutated *ACT1* intron mutated, 3ꞌ splice site mutated *ACT1* intron. A1 and A2 indicate the position of primer pairs used in RT-PCR analysis to monitor splicing. TSS is transcription start site, and TTS indicates transcription termination site. (B) Pre-mRNA of *IMD4* is efficiently spliced in the wild type (WT), but not in splicing defective mutants. Lanes 1 and 3 show RT-PCR results of mRNA of *IMD4* with wild type *ACT1* intron. Lanes 2 and 4 show RT-PCR results of mRNA of *IMD4* with 5ꞌ splice-site (5ꞌ SS) and 3ꞌ splice-site (3ꞌSS) mutations in *ACT1* intron.
(PDF)

S3 Fig **uaRNA and mRNA transcription as well as transcription directionality remains unaffected in a wild type strain at elevated temperature** (A) Schematic depiction of a gene with intron. TSS represents transcription start site, and TTS represents transcription termination site. (B) Quantification of TRO analysis of *IMD4*, *ASC1* and *APE2* in wild type (FY23) at 25°C (black bars) and 37°C (grey bars) to detect the expression of mRNA or uaRNA. The transcript level of 18S was used as a control for normalization. (C) Directionality indices of *IMD4*, *ASC1* and *APE2* in wild type cells at 25°C (black bars) and 37°C (grey bars).
(PDF)

S4 Fig Splicing of pre-mRNA is unaffected in the looping defective strain. (A) Schematic depiction of an intron-containing gene indicating the position of primers used in RT-PCR analysis to monitor splicing. TSS is transcription start site, and TTS indicates transcription termination site. (B) Pre-mRNA of *ASC1 and IMD4* is efficiently spliced in the wild type (WT) and the looping defective *sua7-1* cells. Lane 1 shows PCR of genomic DNA using A1-A2 primers to indicate the size of unspliced RNA. Lanes 2 and 3 show RT-PCR results of mRNA of indicated genes in the wild type and *sua7-1* strains respectively.
(PDF)

S5 Fig Transcription directionality is compromised in the looping defective *sua7-1* strain. (A) Schematic depiction of a gene with intron indicating the sense (mRNA) or the promoter-initiated upstream anti-sense (uaRNA) transcripts. TSS is transcription start site, and TTS indicates transcription termination site. (B) Strand-specific TRO analysis of *IMD4*, *ASC1* and *APE2* in wild type (black bars), and *sua7-1* strain (grey bars) to detect the expression of mRNA or uaRNA. The transcript level of 18S was used as a control for normalization. P values were calculated by two-tailed student t-test. (C) Directionality indices of *IMD4*, *ASC1* and *APE2* in wild type (black bars), and *sua7-1* strain (grey bars).
(PDF)

S1 Table Yeast strains. (PDF)

S2 Table Primers. (PDF)
