# Supplementary figures and images for: Enhancement of Transcription by a Splicing-Competent Intron Is Dependent on Promoter Directionality
Source: PLoS Genet. 2016 May 6;12(5):e1006047. doi: 10.1371/journal.pgen.1006047 (PMC4859611; doi:10.1371/journal.pgen.1006047)

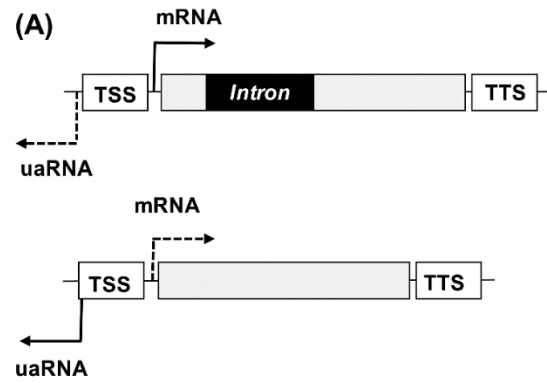

(B)

■ + INTRON □ - INTRON

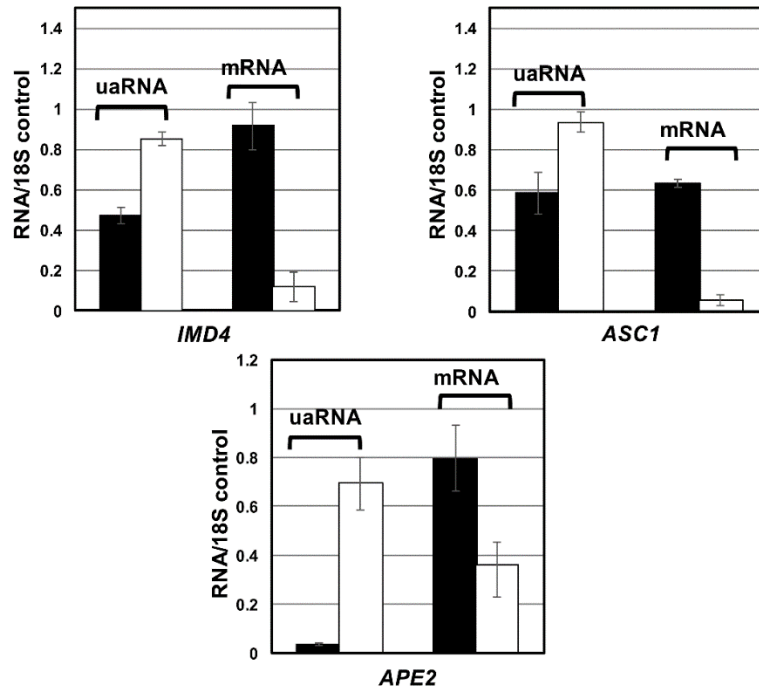

Supplement: S1 Fig — (A) Schematic depiction of a gene with and without intron indicating the sense (mRNA) or the promoter-initiated upstream anti-sense (uaRNA) transcripts. TSS is transcription start site, and TTS indicates transcription termination site. (B) RT-PCR analysis of IMD4, ASC1 and APE2 in the presence (black bars) and absence of intron (white bars) to detect the expression of mRNA or uaRNA. The transcript level of 18S was used as a control for normalization. (PDF) [file pgen.1006047.s001.pdf]

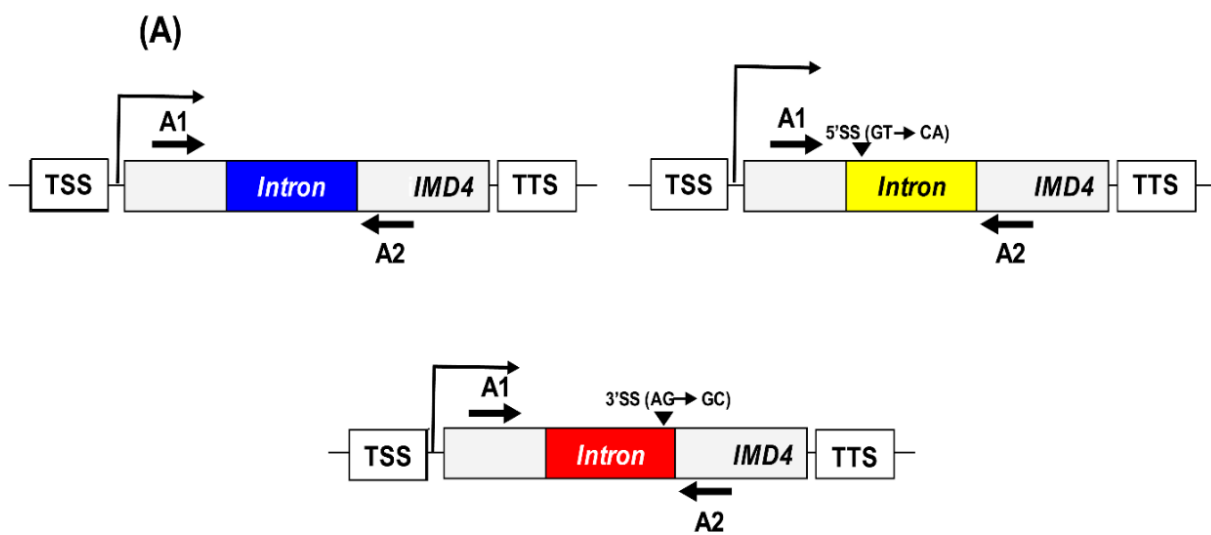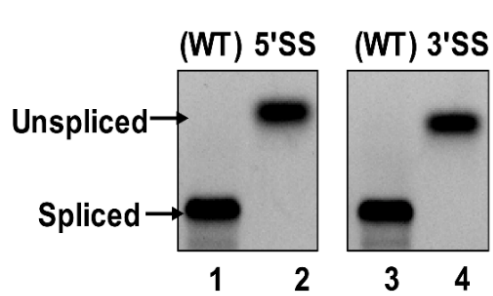

Supplement: S2 Fig — (A) Schematic depiction of IMD4 gene containing wild type ACT1 intron, 5ꞌ splice site mutated ACT1 intron mutated, 3ꞌ splice site mutated ACT1 intron. A1 and A2 indicate the position of primer pairs used in RT-PCR analysis to monitor splicing. TSS is transcription start site, and TTS indicates transcription termination site. (B) Pre-mRNA of IMD4 is efficiently spliced in the wild type (WT), but not in splicing defective mutants. Lanes 1 and 3 show RT-PCR results of mRNA of IMD4 with wild type ACT1 intron. Lanes 2 and 4 show RT-PCR results of mRNA of IMD4 with 5ꞌ splice-site (5ꞌ SS) and 3ꞌ splice-site (3ꞌSS) mutations in ACT1 intron. (PDF) [file pgen.1006047.s002.pdf]

(A)

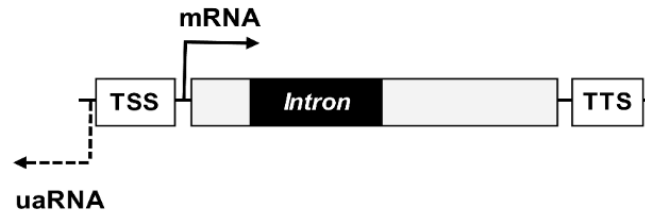

(B)

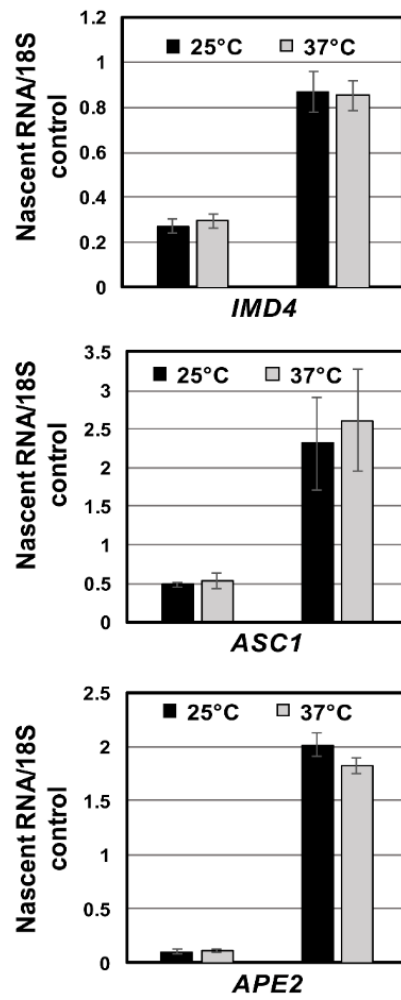

(C)

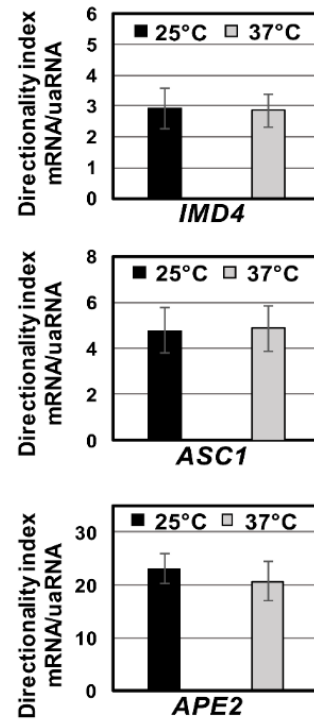

Supplement: S3 Fig — uaRNA and mRNA transcription as well as transcription directionality remains unaffected in a wild type strain at elevated temperature (A) Schematic depiction of a gene with intron. TSS represents transcription start site, and TTS represents transcription termination site. (B) Quantification of TRO analysis of IMD4, ASC1 and APE2 in wild type (FY23) at 25°C (black bars) and 37°C (grey bars) to detect the expression of mRNA or uaRNA. The transcript level of 18S was used as a control for normalization. (C) Directionality indices of IMD4, ASC1 and APE2 in wild type cells at 25°C (black bars) and 37°C (grey bars). (PDF) [file pgen.1006047.s003.pdf]

(A)

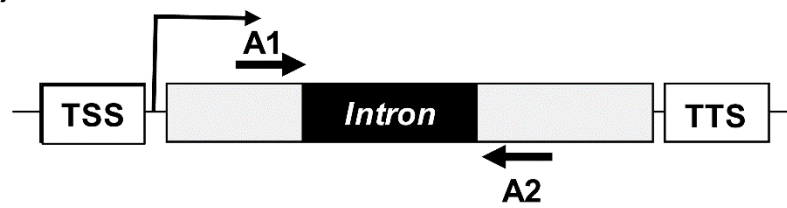

(B)

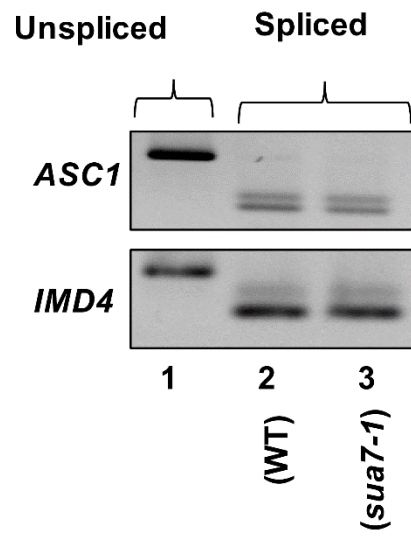

Supplement: S4 Fig — (A) Schematic depiction of an intron-containing gene indicating the position of primers used in RT-PCR analysis to monitor splicing. TSS is transcription start site, and TTS indicates transcription termination site. (B) Pre-mRNA of ASC1 and IMD4 is efficiently spliced in the wild type (WT) and the looping defective sua7-1 cells. Lane 1 shows PCR of genomic DNA using A1-A2 primers to indicate the size of unspliced RNA. Lanes 2 and 3 show RT-PCR results of mRNA of indicated genes in the wild type and sua7-1 strains respectively. (PDF) [file pgen.1006047.s004.pdf]

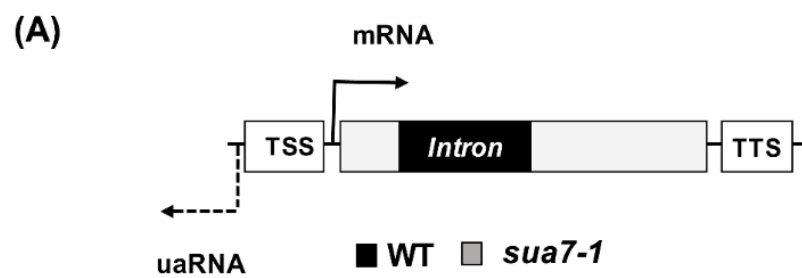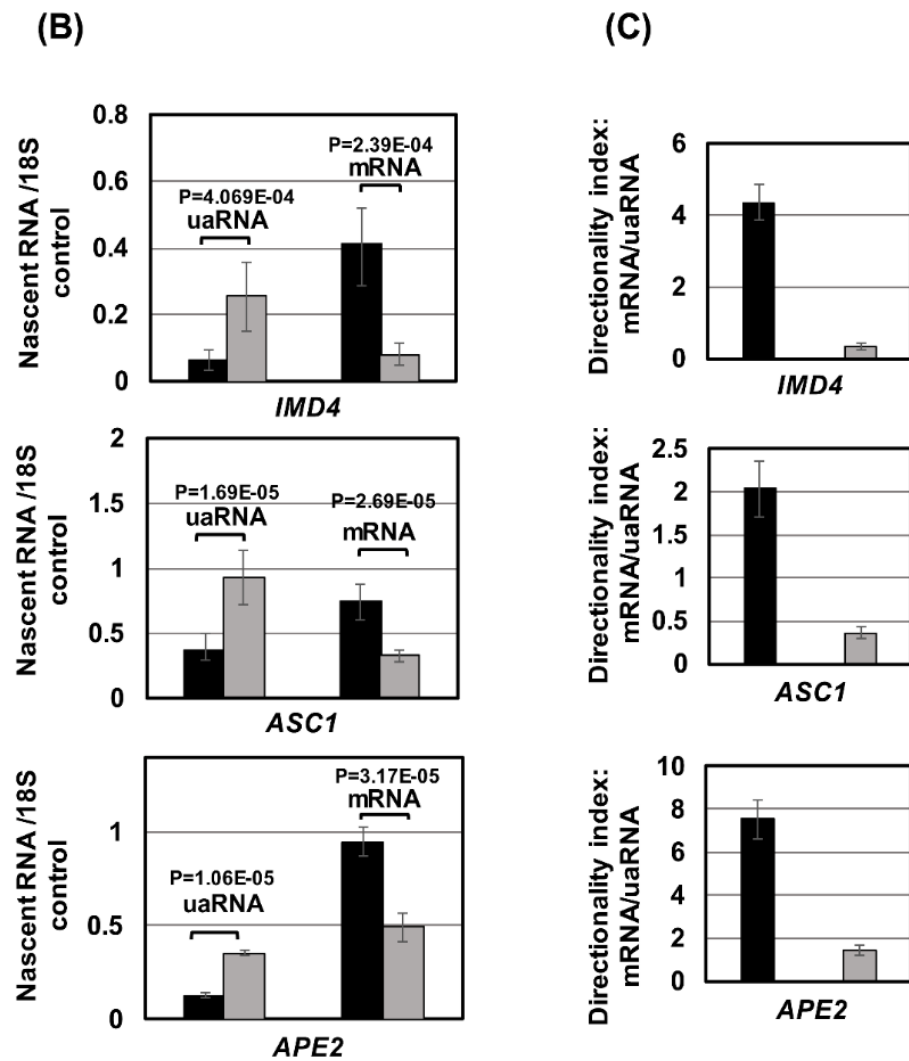

Supplement: S5 Fig — (A) Schematic depiction of a gene with intron indicating the sense (mRNA) or the promoter-initiated upstream anti-sense (uaRNA) transcripts. TSS is transcription start site, and TTS indicates transcription termination site. (B) Strand-specific TRO analysis of IMD4, ASC1 and APE2 in wild type (black bars), and sua7-1 strain (grey bars) to detect the expression of mRNA or uaRNA. The transcript level of 18S was used as a control for normalization. P values were calculated by two-tailed student t-test. (C) Directionality indices of IMD4, ASC1 and APE2 in wild type (black bars), and sua7-1 strain (grey bars). (PDF) [file pgen.1006047.s005.pdf]
