# Supplementary material for: Enhancement of Transcription by a Splicing-Competent Intron Is Dependent on Promoter Directionality
Source: PLoS Genet. 2016 May 6;12(5):e1006047. doi: 10.1371/journal.pgen.1006047 (PMC4859611; doi:10.1371/journal.pgen.1006047)
Supplement: S1 Table — (PDF) [file pgen.1006047.s006.pdf]

S1_Table

Strain	Genotype	Reference
BY4733	*MATa his3Δ200 trp1Δ63 leu2Δ0 met15Δ0 ura3Δ0*	
NEA6	*MATa his3Δ200 trp1Δ63 leu2Δ0 met15Δ0 ura3Δ0 IMD4 Without intron*	This study
NEA10	*MATa his3Δ200 trp1Δ63 leu2Δ0 met15Δ0 ura3Δ0 IMD4::Act1 intron*	This study
AA6	*MATa his3Δ200 trp1Δ63 leu2Δ0 met15Δ0 ura3Δ0 ASC1 Without intron*	This study
HG3	*MATa his3Δ200 trp1Δ63 leu2Δ0 met15Δ0 ura3Δ0 APE2 Without intron*	This study
NEA12	*MATa his3Δ200 trp1Δ63 leu2Δ0 met15Δ0 ura3Δ0 IMD4:: 3'SS (CT→GC) intron*	This study
NEA13	*MATa his3Δ200 trp1Δ63 leu2Δ0 met15Δ0 ura3Δ0 IMD4 Without intron,RNA15-TAP::TRP1*	This study
ANN1	*MATa his3Δ200 leu2Δ0 met15Δ0 ura3Δ0 IMD4 ,NAB3-TAP::TRP1*	This study
ANN2	*MATa his3Δ200 trp1Δ63 leu2Δ0 met15Δ0 ura3Δ0 IMD4 Without intron, NAB3-TAP::TRP1*	This study
ANN3	*MATa his3Δ200 trp1Δ63 leu2Δ0 met15Δ0 ura3Δ0 IMD4 Without intron, RAT1-TAP::TRP1*	This study
ZD7	*MATa his3Δ200 trp1Δ63 leu2Δ0 met15Δ0 ura3Δ0, RAT1-TAP::TRP1*	This study
KMD1	*MATa his3Δ200 trp1Δ63 leu2Δ0 met15Δ0 ura3Δ0 ASC1 Without intron, RAT1-TAP::URA3*	This study
ANN4	*MATa his3Δ200 trp1Δ63 leu2Δ0 met15Δ0 ura3Δ0 IMD4 Without intron, PTA1-HA::HIS3*	This study
SAM72	*MATa his3Δ200 trp1Δ63 leu2Δ0 met15Δ0 ura3Δ0 ASC1 without intron,RNA15-TAP::URA3*	This study

KMD2	*MATa his3Δ200 trp1Δ63 leu2Δ0 met15Δ0 ura3Δ0 ASC1 without intron,RNA15-TAP::URA3*	This study
NEA17	*MATa his3Δ200 trp1Δ63 leu2Δ0 met15Δ0 ura3Δ0 IMD4:: 5'SS (GT→CA) intron*	This study
KMD3	*MATa his3Δ200 trp1Δ63 leu2Δ0 met15Δ0 ura3Δ0 ASC1 without intron, PTA1-HA::HIS3*	This study
KMD4	*MATa his3Δ200 trp1Δ63 leu2Δ0 met15Δ0 ura3Δ0 ASC1 without intron, NAB3-TAP::URA3*	This study
AS1	*MATa his3Δ200 trp1Δ63 leu2Δ0 met15Δ0 ura3Δ0 sua7-1*	This study
NEA19	*MATa his3Δ200 trp1Δ63 leu2Δ0 met15Δ0 ura3Δ0 sua7-1, PTA1-HA::HIS3*	This study
NEA20	*MATa his3Δ200 trp1Δ63 leu2Δ0 met15Δ0 ura3Δ0 sua7-1, RAT1-TAP::TRP1*	This study
KMD5	*MATa his3Δ200 trp1Δ63 leu2Δ0 met15Δ0 ura3Δ0 sua7-1, RNA15-TAP::TRP1*	This study
ANN5	*MATa his3Δ200 trp1Δ63 leu2Δ0 met15Δ0 ura3Δ0 IMD4:: 5' SS (GT→CA) intron,PTA1-HA::HIS3*	This study
ANN6	*MATa his3Δ200 trp1Δ63 leu2Δ0 met15Δ0 ura3Δ0 IMD4:: 3' SS (CT→GC) intron,PTA1-HA::KANMX*	This study
SAM51	*MATa his3Δ200 trp1Δ63 leu2Δ0 met15Δ0 ura3Δ0, RNA15-MYC*	This study
KMD7	*MATa his3Δ200 trp1Δ63 leu2Δ0 met15Δ0 ura3Δ0 IMD4:: 5' SS (GT→CA) intron,RNA15-MYC::KANMX*	This study
KMD8	*MATa his3Δ200 trp1Δ63 leu2Δ0 met15Δ0 ura3Δ0 IMD4:: 3' SS (CT→GC) intron,RNA5-MYC::KANMX*	This study
FY23	*MATa ura3-52 trp1D63 leu2D1*	*Winston et al. (1)*
rna15-2	*MATa ura3-1 trp1-1 ade2-1 leu2-3,112 his3-11,15*	*Minvielle-Sebastia L et al. (2)*

XHy23	*Pta1-degron construct: CUP1p-Ub-Arg-DHFRts-HA-PTA1--URA3*	*Krishnamurthy et al. (3)*

References:

1. Madison JM, Winston F. Evidence that Spt3 functionally interacts with Mot1, TFIIA, and TATA-binding protein to confer promoter-specific transcriptional control in Saccharomyces cerevisiae. Mol Cell Biol. 1997;17(1):287-95.
2. Minvielle-Sebastia L, Winsor B, Bonneaud N, Lacroute F. Mutations in the yeast RNA14 and RNA15 genes result in an abnormal mRNA decay rate; sequence analysis reveals an RNA-binding domain in the RNA15 protein. Mol Cell Biol. 1991;11(6):3075-87.
3. Krishnamurthy S, He X, Reyes-Reyes M, Moore C, Hampsey M. Ssu72 Is an RNA polymerase II CTD phosphatase. Mol Cell. 2004;14(3):387-94.
