# Supplementary material for: Enhancement of Transcription by a Splicing-Competent Intron Is Dependent on Promoter Directionality
Source: PLoS Genet. 2016 May 6;12(5):e1006047. doi: 10.1371/journal.pgen.1006047 (PMC4859611; doi:10.1371/journal.pgen.1006047)
Supplement: S2 Table — (PDF) [file pgen.1006047.s007.pdf]

S2_Table

ChIP Primers

Name	Sequence
IMD4 A	GCCATATAAATATCAGTTGAGAATCC
	GTATGTCTTCAAATGTTCTAAAGCC
IMD4 B	AGTTTGGTTTCTCTGGCTTCC
	GATCACAGTCAGTAGAACGAAAGTT
IMD4 C	AACTTTCGTTCTACTGACTGTGATC
	CTCAACTTTATGAAGACTGAAGAAAT
IMD4 D	ATTTGATGAAGAATCAAAACTACCC
	AAATCTGGGAAAGTTTCTTTAATCC
IMD4 E	GAGTAAGCATCCATAGATATTTAAAAG
	CGAACTGAAAAACGAAAATAAGAA
ASC1 A	GACTGCTCCTTTGGTTTTCC
	GGTTGACCAGCAGAAGGAGCC
ASC1 B	GCTTCCATGATTATCTCTGGT
	CTCGATTGTCATCATATTCTATCA
ASC1 C	CTTCTCTTTATCCGTTATGTCAAAATG
	TTCTTAGCAGCCAAGTTCCACA
ASC1 D	TTGGCTGCTAAGAAGGCTATG
	CCAAGCCAAAGAAACAGCAT
ASC1 E	GTTTCTTTGGCTTGGTCTGC
	GCCAAGGAGACTGAATTTAATG

cDNA primers for TRO and Strand-specific RT PCR:

Oligo dT	TTTTTTTTTTTTTTTTTTTTTTTTT
18S	GACGGAGTTTCACAAGATTACC
IMD4 UP1	GATTCAAATCATGCTTGGCTC

ASC1 UP1	ACTGGTAATACTCATCACCATACTTATAT
APE2 UP1	GCGGCAAGATAGTAAGATAGCA

Upstream antisense RT PCR primers:

IMD4 UP	CGCATTTTTTCATCTCTTTTTC
	TTTGTTAGGTTTCTTCAAAGCTATG
ASC1 UP	GTGCTTCTCCAGCGAAAGTC
	CACAATTAAAGGAATAGCCCAA
APE2 UP	TGAACCTTACAGCGCCTT
	GTCTAAGAGCACATTAGATCGAA

Gene specific (mRNA) RT primer:

IMD4 A1	TGTTGTCAAGAGCGGATTTG
IMD4 A2	AATCTGGGAAAGTTTCTTTAATCC
ASC1 A1	TTGGCTGCTAAGAAGGCTATG
ASC1 A2	CGGTAGCAGTGGCAGCAG
APE2 A1	CCAATTGTTCGGTGGCTATT
APE2 A2	ACAAATCTTGGGGGAGTTAATT
18S F	GGAATAATAGAATAGGACGTTTGG
18s R	GTTAAGGTCTCGTTCGTTATCG

CCC primer:

IMD4 P1	AGAGTTTTTCACATTAGGGCTGC
IMD4 T1	CTTATTGAAGTATGTACAGTGGAAATAG
IMD4 F1	TGGATTACAAAAAGGCTTTAGAAC
IMD4 R1	CTTCAGTGACTGTGTCCATAGGAG
ASC1 P1	AGGAAAACCAAAGGAGCAGTC
ASC1 T1	TGGCAAGTTATGACTGCTAACTAAG

ASC1 F1	TTGGCTGCTAAGAAGGCTATG
ASC1 R1	CGGTAGCAGTGGCAGCAG
APE2 P1	CAGTTTAGAAGTTTACCAAACC
APE2 T1	ATTTTGCCTTTTTATATAGTCAAGT
APE2 F1	ATAATATGGCTGGCTTTTACA
APE2 R1	TAAGTTGACATTTTAGGGGTC
